# Efficacy of Vitamin D Supplementation on the Risk of Falls Among Community-Dwelling Older Adults: A Systematic Review and Meta-Analysis

**DOI:** 10.3390/jcm14176117

**Published:** 2025-08-29

**Authors:** Raquel Torres-Lopez, Núria Obradors, Roberto Elosua, Rafael Azagra-Ledesma, Marta Zwart

**Affiliations:** 1 Doctoral Program in Medicine and Biomedical Sciences, University of Vic-Central University of Catalonia (UVic-UCC), Sagrada Familia Street, 7, 08500 Vic, Barcelona, Spain; raquel.torres1@uvic.cat; 2 Research Group on Osteoporosis, Falls and Musculoskeletal Pathology in Primary Care (GROICAP), Research Support Unit (USR), Girona-IDIAP Jordi Gol, Maluquer Salvador Street, 11, 17002 Girona, Spain; marta.zwart@udg.edu; 3 Health Center Badia del Vallès, Institut Català de la Salut (ICS), Bètica Street s/n, 08214 Badia del Vallès, Barcelona, Spain; 4 Faculty of Health Sciences and Welfare, University of Vic-Central University of Catalonia (UVic-UCC), Sagrada Família Street, 7, 08500 Vic, Barcelona, Spain; 5 Tissue Repair and Regeneration Laboratory (TR2Lab), Institute for Research and Innovation in Life and Health Sciences in Central Catalonia (IrisCC), Faculty of Medicine, University of Vic-Central University of Catalonia (UVic-UCC), Roda Road 71, 08500 Vic, Barcelona, Spain; nuria.obradors@uvic.cat; 6 Faculty of Medicine, University of Vic-Central University of Catalonia (UVic-UCC), Roda Road 71, 08500 Vic, Barcelona, Spain; roberto.elosua@umedicina.cat; 7 Cardiovascular Epidemiology and Genetics Group (EGEC), Hospital del Mar Research Institute, Doctor Aiguader Street 88, 08003 Barcelona, Spain; 8 Cardiovascular Disease Biomedical Research Network Center (CIBERCV), Monforte de Lemos Street 3–5, 28029 Madrid, Spain; 9 PRECIOSA Private Foundation for Research, Comadran Street 9, 08210 Barberà del Vallès, Barcelona, Spain; 10 Unitat de Coordinació d’Estudiants de Grau, Unitat Docent Multiprofesional, Gerencia d’Atencio Primaria i a la Comunitat (GAPIC) Barcelones Nord i Maresme i GAPIC Vallès, Institut Català de la Salut, Generalitat de Catalunya, Velazquez Square s/n, 08290 Cerdanyola, Barcelona, Spain; 11 Department of Medicine, Universitat Autònoma de Barcelona, Edifici M, Av. de CanDomenech, 08193 Bellaterra, Barcelona, Spain; 12 Family Medicine, Health Center Can Gibert del Pla, Institut Català de la Salut (ICS), San Sebastián Street 9, 17005 Girona, Spain; 13 Medical Sciences Department, Girona University (UdG), Emili Grahit Street 77, Center Campus, 17003 Girona, Spain

**Keywords:** vitamin D (VitD), aging, falls, meta-analysis

## Abstract

**Background/Objectives**: Previous meta-analyses on vitamin D (VitD) supplementation for fall prevention have mixed different populations and doses. This study aimed to evaluate whether VitD supplementation reduces fall risk in community-dwelling adults aged ≥65 years. **Methods**: Randomized clinical trials (RCTs) including adults ≥65 years living in the community and receiving supplemental VitD were identified through the MEDLINE and EMBASE databases (January 2005–July 2024), and independent reviewers selected studies reporting fall risk, extracted data, and assessed risk of bias. Outcomes were pooled using the inverse variance method. Heterogeneity and publication bias were assessed with I^2^, Egger’s test, and the trim-and-fill method. **Results**: The analysis dataset was 23,211 participants from 10 RCTs. Most studies had minimal risk of bias. Overall, VitD supplementation was not associated with a reduction in fall risk, as no statistically significant or consistent trend was observed (OR = 0.99; 95%CI: 0.95–1.03; I^2^ = 31%). In women, no significant association was found (OR = 0.97; 95%CI: 0.92–1.02; I^2^ = 31.2%), and in men, no significant association was observed (OR = 1.08; 95%CI: 0.98–1.20; I^2^ = 0%) when analyzed separately. Supplementation with doses ≤1000 IU/day showed no significant association with fall risk (OR = 0.96; 95%CI: 0.90–1.02; I^2^ = 39.5%), as did durations ≤12 months (OR = 0.96; 95%CI: 0.90–1.02; I^2^ = 56.2%) and daily administration (OR = 0.97; 95%CI: 0.92–1.03; I^2^ = 37.2%). Doses >1000 IU/day, intermittent dosing (both OR = 1.02; 95%CI: 0.96–1.09; I^2^ = 0%), and durations >12 months (OR = 1.01; 95%CI: 0.96–1.07; I^2^ = 0%) all showed no significant association. Although evidence of publication bias was detected, adjustment did not alter the results. **Conclusions**: This meta-analysis shows that VitD supplementation has no effect on the risk of falls in community-dwelling adults ≥65 years, yet its main interest lies in ensuring population homogeneity found in previous analyses of mixed settings.

## 1. Introduction

Various epidemiological studies show that 30% of adults experience a fall each year, particularly if they are functionally frail or institutionalized, and that the aging of the population increases the magnitude of the problem [1,2,3]. Falls are the second leading cause of death from accidental trauma, result in hospital admission for fractures and home or residential confinement, and limit quality of life [4]. Fear of falling, though perhaps causing no immediate physical harm, can lead to people reducing their physical activity, thus increasing the risk of falls [5]. Prevention should address both aspects with strategies that encourage mobility [3,6]. Studies on fall prevention focus on single interventions or multifactorial programs [3,6,7]. These include physical activity, home-safety advice, a balanced diet, suitable footwear, correction of vision problems, and vitamin D (VitD) supplementation [8]. Results, however, do not always clearly demonstrate their effectiveness.

Uncertainties and controversies surround the issue of the efficacy of VitD supplements. Firstly, there is no consensus on the blood cholecalciferol level at which supplementation is indicated [9,10,11,12,13]. Moreover, the J-shaped relationship between supplementation and risk of falls (low doses have no significant impact, moderate doses reduce risk, and high doses increase it) makes interpretation difficult and underlines the importance of appropriate dosage [14]. Finally, recommendations vary according to factors such as gender, climate, comorbidities, and health policies [9,10,11]. Regarding gender, evidence indicates that supplementation reduces the risk of falls in women but not in men [15]. This is relevant as women, with lower bone density and muscle mass, are at greater risk of fractures due to frailty [16]. Regarding climate, some studies indicate that supplementation is more effective in winter, which calls into question the need for seasonal prevention [17]. In summary, the assessment of the preventive effect of VitD on falls is a complex field requiring the consideration of multiple variables. Previously published meta-analyses have included highly diverse populations, spanning different backgrounds and age groups, which increases heterogeneity by combining participants from community settings, outpatient clinics, hospitals, and residential care facilities. Therefore, the aim of this study is to evaluate whether VitD supplementation improves fall prevention in a specific population group of people over 65 years of age living in a community setting.

## 2. Materials and Methods

### 2.1. Design

This study is a systematic review and meta-analysis. It was registered in the PROSPERO database (CRD42023417763) [18], followed the *Cochrane Handbook for Systematic Reviews of Intervention v5.1.0* [19], and was reported in accordance with the Preferred Reporting Items for Systematic Reviews and Meta-Analysis (PRISMA) guidelines [20].

### 2.2. Search Strategy

Two databases—MEDLINE and EMBASE—were searched using a predefined strategy, detailed in Table S1. The search period covered 1 January 2005 to 29 July 2024. To guide the systematic review, the research question was formulated using the Population, Intervention, Comparator, Outcome, Study design, and Time frame (PICOST) format, as follows:

P (Population): individuals over 65 years residing in the community;
I (intervention): VitD supplementation;
C (Comparison or control): placebo;
O (Outcomes): fall;
S (Study design): randomized clinical trial;
T (Time frame): January 2005 to July 2024.

Inclusion criteria: randomized clinical trials that adequately defined the intervention with VitD supplementation and reported data allowing fall outcomes to be calculated.

Exclusion criteria: studies published in languages other than English or Spanish and those involving subjects living in nursing homes.

### 2.3. Data Extraction

Three reviewers independently screened articles by title and abstract, and two reviewers conducted the full-text assessments. Discrepancies were resolved by consensus. For each selected article, the bibliography was carefully examined to identify any relevant studies not captured by the predefined search strategy. Grey literature was not searched. Data collected included lead author, journal and year of publication, country, number of patients, proportions of males and females, mean age, follow-up period, VitD/calcium supplementation (details on dose, frequency, and duration), comparator groups, and fall frequencies. Data management was performed using the Rayyan application version 1.4.3.

### 2.4. Effect Size of the Association of Interest

The Odds Ratio (OR), Relative Risk (RR), or Hazard Ratio (HR) and its 95% Confidence Interval (CI) of the association between VitD supplementation, alone or plus calcium, and the risk of falls, were registered. In the case of results at different follow-up times, the longest follow-up was selected.

### 2.5. Analysis of the Risk of Bias

Risk of bias analysis was performed using the Cochrane Risk of Bias (RoB 2) tool [21]. RoB 2 is structured into five domains of bias (randomization process, deviation from the intended interventions, missing outcome data, measurement of the outcome, and selection of the reported results) and provides an overall assessment of the risk of bias for each study. Within each domain, a series of questions aims to elicit information about features of the trial that are relevant to risk of bias. A proposed judgement about the risk of bias arising from each domain is generated by an algorithm based on answers to the signaling questions. Judgement can be ‘Low’ or ‘High’ risk of bias or can express ‘Some concerns’. A GRADE assessment was also performed to evaluate the certainty of evidence [22].

### 2.6. Statistical Analysis

A meta-analysis was conducted, and the summary estimates were obtained using the inverse variance weighting method. Both fixed- and random-effects models were applied to assign weights to each study, with the results chosen according to the level of heterogeneity. Heterogeneity between studies was assessed using the I^2^ statistic. The fixed-effect model results were selected when heterogeneity was low (I^2^ < 50%) and the random-effects model results when heterogeneity was moderate or high (I^2^ ≥ 50%). The presence of possible publication bias was evaluated using the funnel plot test and the Egger’s test. The trim-and-fill method was used to account for potential publication bias [23].

Several sensitivity analyses were also conducted: stratification by sex, VitD dose (≤1000 vs. >1000 IU), dosing regimen (daily vs. intermittent), baseline VitD levels (deficient vs. normal), and treatment duration (≤12 vs. >12 months). Moreover, a leave-one-out sensitivity analysis was also performed. Analyses were performed using the ‘meta’ package of R-Studio version 4.4.1.

## 3. Results

### 3.1. Study Search and Characteristics

**Figure 1:**
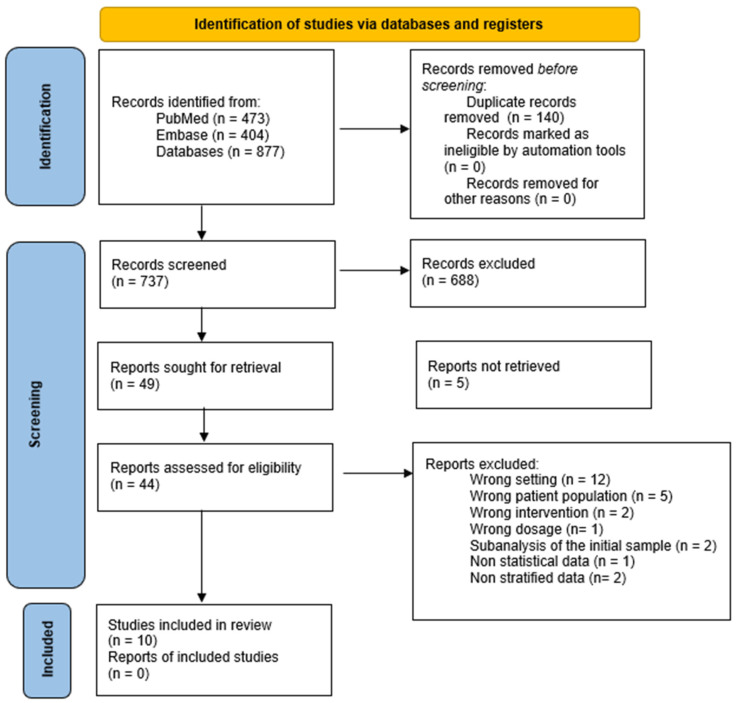
Descriptive flow chart of the selection process for randomized clinical studies.

Figure 1 summarizes the literature search, data extraction, and management of the study selection. Ten studies were included after title, abstract, and full-text screening. Baseline characteristics are presented in Table 1. Of the total, 50% of the studies were performed in Europe, 20% in North America, and 30% in Oceania. Of a total population of 23,211, 13,510 were women and 9701 men, aged 74.24 ± 4.6 years old, living in the community, and two articles were in homebound individuals. Nine studies used cholecalciferol and one ergocalciferol. The dose of VitD used in most studies was high, between 700 and 2000 IU/day. In some doses, supplementation was monthly or quarterly, but in none did it exceed 3500 IU/day (Table S2). The quality of the meta-analysis articles is reported in Table S3.

Table S4 shows the reason for exclusion for each study (reports assessed for eligibility n = 34).

### 3.2. Findings (Results) of the Individual Studies

Across the included trials, the results were heterogeneous. Among studies using daily dosing in community-dwelling adults, Bischoff-Ferrari et al. [15] reported a non-significant 33% reduction in falls overall and a significant 46% reduction in women, with no benefit in men and an increased risk among the most active women. Prince et al. [24] found a non-significant 39% reduction in women, with a significant 45% reduction in winter/spring but no effect in summer/autumn. Pfeifer et al. [25] observed a statistically significant 27% reduction in falls compared with the control group. Kärkkäinen et al. [26] reported a non-significant 2% reduction overall but significant reductions for multiple falls (30%) and medically attended falls (28%). Appel et al. [30] found no benefit for mild falls but a significant doubling of risk for severe falls (HR = 1.87, 95%CI: 1.03–3.41) or those requiring hospitalization (HR = 2.48, 95%CI: 1.13–5.46). Bischoff-Ferrari et al. [32] found no benefit from VitD plus exercise or high-dose VitD. Among studies using intermittent dosing, Glendenning et al. [27] observed a non-significant 6% increase in falls with a single high dose of VitD administered every three months, and Waterhouse et al. [31] showed no overall effect but increased significant risk by 25% in participants with BMI < 25 kg/m^2^ receiving monthly doses. Two studies were conducted in homebound populations. In the first, with daily dosing, Uusi-Rasi et al. [28] found no overall differences in fall incidence between the VitD supplementation and control groups. However, when a structured exercise program was implemented, a significant reduction in falls was observed, 62% in the VitD plus exercise group versus 53% in the exercise-only group, suggesting that physical activity rather than VitD was the main driver of the benefit. In the second study, Houston et al. [29], who administered high monthly doses of VitD, reported a non-significant 52% reduction overall, becoming significant (59%) among participants assessed in winter and with baseline serum 25(OH)D levels below 20.9 nmol/L.

### 3.3. Vitamin D Supplementation Efficacy Results

The effectiveness of VitD supplementation in each study is reported separately for men, women, or the total sample when available. The results are presented as Odds Ratios (OR) with 95% Confidence Intervals, together with beta coefficients and standard errors (lower and upper bounds) (Table S5).

**Figure 2:**
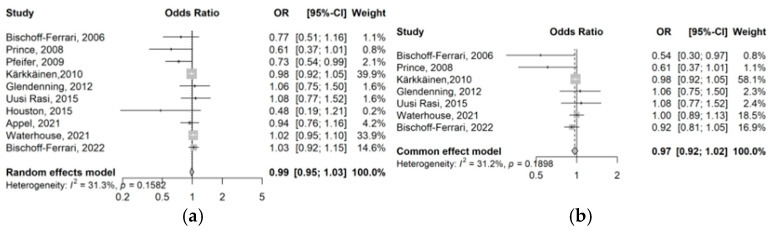

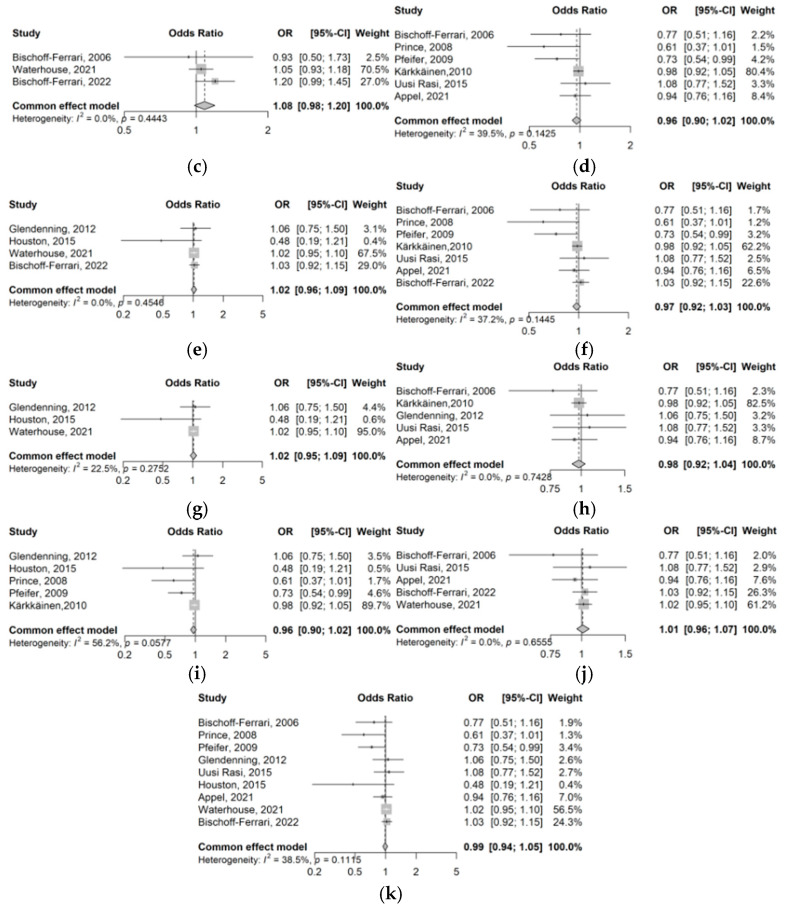
Forest plots of the association of VitD supplementation for falls [15,24,25,26,27,28,29,30,31,32]: (**a**) overall, (**b**) women, (**c**) men, (**d**) ≤1000 IU doses, (**e**) >1000 IU doses, (**f**) daily doses, (**g**) intermittent doses, (**h**) normal baseline VitD levels, (**i**) ≤12 months, and (**j**) >12 months, (**k**) Without calcium supplementation. OR: Odds Ratio. CI: 95% Confidence Interval. *p*: *p*-value. I^2^: heterogeneity statistics.

The forest plots showing the effect of VitD supplementation on the risk of falls in the overall analysis and in the various sub-analyses are shown in Figure 2 and Table 2. Heterogeneity across study results was low, as indicated by an I^2^ value of less than 50% and a *p*-value greater than 0.05.

The results of the meta-analysis that included both sexes combined showed a tendency toward a possible effect in the likelihood of falls (OR = 0.99; 95%CI 0.95–1.03, I^2^ = 31.3%, *p* = 0.16). The analysis of studies solely in women revealed similar results (OR = 0.97, 95%CI 0.92–1.02, I^2^ = 31.2%, *p* = 0.19) while those in men showed non-significant increases (OR = 1.08, 95%CI 0.98–1.20, I^2^ = 0%, *p* = 0.44).

The results of the meta-analysis including only doses ≤ 1000 UI/day showed a non-significant reduction in risk (OR = 0.96, 95%CI 0.90–1.02, I^2^ = 39.5%, *p* = 0.14). In contrast, studies with doses >1000 IU/day reported a non-significant increase (OR = 1.02, 95%CI 0.96–1.09, I^2^ = 0%, *p* = 0.46).

The meta-analysis of studies with daily VitD supplementation showed a non-significant reduction in risk (OR = 0.97, 95%CI 0.92–1.03, I^2^ = 37.2%, *p* = 0.14), while high-dose intermittent supplementation (monthly or quarterly) revealed a non-significant increase (OR = 1.02, 95%CI 0.95–1.09, I^2^ = 22.5%, *p* = 0.27).

The meta-analysis of the five studies with normal VitD baseline levels [10,22,24,26,30] indicated a non-significant reduction in risk (OR = 0.98, 95%CI 0.92–1.04, I^2^ = 0.0%, *p* = 0.74).

The meta-analysis of studies of a duration ≤ 12 months presented a non-significant risk reduction (OR = 0.96, 95%CI 0.90–1.02, I^2^ = 56.2%, *p* = 0.06), while those studies > 12 months (OR = 1.01, 95%CI 0.96–1.07, I^2^ = 0.0%; *p* = 0.66) revealed a non-significant increase.

### 3.4. Publication Bias and Sensitivity Analysis

The funnel plot (Figure S1) showed overall symmetry between studies, with data points concentrated around the upper center line, although some deviations were noted in the lower portion. A sensitivity analysis was performed to assess how the exclusion of individual studies affected the consistency of the overall results, and it was observed that, apart from one [26], the results were robust. Beta coefficients and *p*-values were consistent for the fixed and random models, as was statistical significance (Table S6). Egger’s test produced consistent values across common and random-effects models, suggesting minimal heterogeneity in the included studies. Egger’s test indicated that there was statistically significant asymmetry in the funnel plot (t = −2.46, df = 8, *p* = 0.0395), which raises the possibility of publication bias. The intercept was estimated at −1.772 (SE = 0.4791), suggesting that smaller studies may report systematically different effect sizes than larger ones. Finally, the residual heterogeneity variance (T2) was 0.9332, pointing to a moderate level of variability across the included studies. The trim-and-fill method included two additional studies, and the overall effect was similar to the original pooled estimate (OR = 0.99; 95%CI 0.95–1.03; *p* = 0.058). This shows that there is no evidence of an association between falls and VitD supplementation. This result is robust even after correcting for possible publication bias (Figures S2 and S3).

### 3.5. Risk of Bias and GRADE Assessment of the Evidence

**Figure 3:**
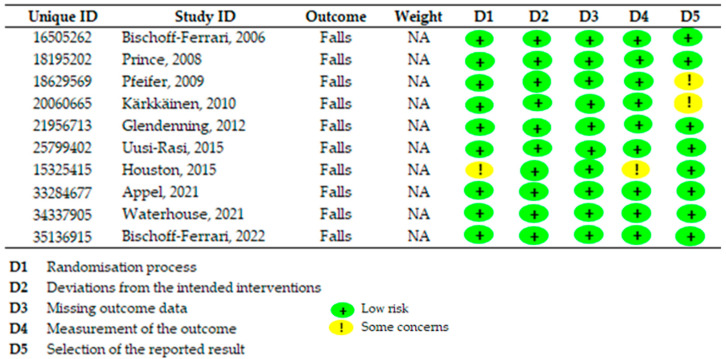
Evaluation of the presence of bias in the selected articles using the Cochrane Risk of Bias Tool scale [15,24,25,26,27,28,29,30,31,32].

Figure 3 presents the risk of bias summary, showing minimal risk of bias in most studies, except for some concerns in three studies. Pfeifer et al. lacked a prior data analysis plan, and results for men and women were combined [25]. Kärkkäinen et al. did not conceal allocation, potentially influencing the control group’s dietary behavior [26]. Finally, Houston et al. raised concerns regarding bias in the randomization process [29]. No study showed a high risk of bias.

The GRADE assessment indicated that the evidence was derived from studies with a low risk of bias. Moreover, issues related to inconsistency, indirectness, and imprecision appeared to be minimal, and potential publication bias was considered to have only a minor impact on the overall effect estimate. Accordingly, the quality of the evidence supporting the lack of an effect of VitD supplementation on fall risk was considered to be moderate to high.

## 4. Discussion

The results of the meta-analysis with 23,211 participants (53% women) showed a non-significant tendency regarding the incidence of falls with VitD supplementation. Six of the studies showed an OR < 1, with only one significant [25], although small robust reductions can have important implications in patient subgroups. It is conceivable that in a larger sample size they could be significant. The funnel plot illustrates that there is a scarcity of studies in the lower part and the possible presence of publication bias from certain smaller studies. While such bias was detected, its impact on the pooled estimates appeared minimal. The plot also indicated a possible lack of unpublished studies with null effects and large standard errors. However, imputing these using the trim-and-fill method did not materially change the results.

Participant characteristics may also have influenced results (I^2^ = 31.3%), such as the inclusion of women without severe comorbidities [26], only those who were able to exercise [24], or with a history of falls [24,28]. In addition, inadequate adherence leading to effect inconsistency could be a further cause [24]. Regional diversity introduces variability due to geographical and demographic factors: darker skin, latitude altering exposure to the sun, altitude, degree of pollution, and hours of exposure are factors to consider and are present in the meta-analysis [15,25,26,28,32]. There are also differences in the recording of falls: some are carried out daily [24,25,27,28,30,31], others by letter after each fall [15,32], and even those without the need for a record involving a call every four months [26] or monthly [29]. Less exhaustive methods may underestimate falls. It should be noted that calcium was administered in four of the studies, to both groups in two [24,25] and to the intervention group only in two [15,26], making it difficult to distinguish between the effect of the VitD, the calcium, or their combination. However, an analysis excluding these four trials showed no significant effect, indicating that calcium co-administration is unlikely to have influenced the overall results. Previous meta-analyses reported significant and non-significant results. Among the latter, Jackson et al. (5 RCTs; 3776 participants) reported a non-significant reduction of 12% (I^2^ = 8.3%) [33]. Among the former, Thanapluetiwong et al. (47 RCTs; 58,424 participants) [34], Kong et al. (32 RCTs; 104,363 participants) [35], Murad et al. (26 RCTs; 45,782 participants) [12], and Wei et al. (38 RCTs; 61,350 participants) [36] found significant reductions of 5.2% (I^2^ = 41.52%), 9% (I^2^ = 69.8%), 14% (I^2^ = 66%), and 13% (I^2^ = 80%), respectively. These studies show greater heterogeneity than the present study (I^2^ = 31.3%). That is, they combined populations from the community and outpatient, hospital, and residential care, introducing variability in the baseline risk of falls, as shown in a study with a reduction of 49% among patients after supplementation in an exclusively institutionalized setting [37].

Sensitivity analyses by sex showed a tendency toward a reduction in falls among women and an increase in men, although neither reached statistical significance. Only one study showed a significant reduction of 46% in women following 3 years of supplementation, while in men it was not significant [15]. These findings are consistent with studies indicating that women have poorer baseline measurements of strength, gait, and balance, which would make them more receptive to supplementation [16]. Furthermore, older women more frequently present with VitD deficiency and postmenopausal hormonal changes, which may exacerbate functional deficits and provide greater potential for measurable improvement with VitD supplementation compared with men. In addition, a greater benefit was observed in less active women, suggesting that supplementation is more effective in vulnerable populations. Nevertheless, other studies report conflicting results, with no influence on functional decline [38]. In previous meta-analyses, Murad et al. [12], Bischoff-Ferrari et al. (8 RCTs; 2426 participants) [39], Jackson et al. [33], and Wei et al. [36] estimated a tendency towards reduction in women of 22%, 15%, 8%, and 13%, respectively, although none reached significance. Shifting back to the analyses including both sexes, Tan et al. (35 RCTs; 58,937 participants) [13] and Thanapluetiwong et al. [34] found significant reductions of 22% in women and 8.3% in men, respectively.

Regarding the doses of VitD supplementation, administered daily or intermittently, there are differences that may affect the interpretation of results: although the daily doses present greater heterogeneity, with variability possibly attributed to the doses used (800–1000 UI/day, except one study with 2000 UI/day), they show a generally favorable effect, with a reduction of 3% in the risk of falls, while intermittent doses are associated with an increase of 2%, with neither reaching significance. When comparing doses of ≤1000 IU/day, in line with current ESPEN expert group recommendations for daily trace element and vitamin intake, a reduction of 4% is observed, while doses of >1000 IU/day are associated with an increase of 2%, with neither being significant [40]. Prior meta-analyses reported results that were more consistent with daily supplementation, such as Thanapluetiwong et al., with a significant reduction of 8.1%, but similar results were shown with intermittent high doses, suggesting that they may increase risk rather than offering additional protection [34]. Zheng et al. (9 RCTs; 22,012 participants) also reported excess risk [41]. Two meta-analyses assessed efficacy at specific doses of between 700 and 1000 UI/day, with significant results: Tan et al. (35 RCTs; 58,937 participants) showed a reduction of 15% (I^2^ = 11%) [13] and Bischoff-Ferrari et al. (7 RCTs; 1921 participants) 19% (I^2^ = 41%) [39]. The sub-analysis of doses of ≤1000 IU/day in this meta-analysis, although administered in a homogeneous community-dwelling population, showed an I^2^ value of 39.5%, suggesting that other factors in addition to setting and dosage could have contributed to the variability. For instance, in one study, participants received ergocalciferol, and two studies were conducted in the subject of receiving home care.

With respect to the efficacy sub-analysis by VitD baseline level, populations that may have responded differently to supplementation were mixed. The forest plot of the five studies with normal baseline levels showed a non-significant reduction of 2% in the risk of falls. Studies with deficiency could not be stratified, as only one study included participants with insufficiency [31], while the remaining studies included both VitD-sufficient and -insufficient subjects [25,29,32]. Nor did other previous meta-analyses find benefits related to sufficiency levels, including Tan et al. [13], although a significant reduction of 31% was found with deficiency levels, along with Murad et al. [12], Ling et al. [42] (31 RCTs; 57,867 participants), and Prince et al. [24] reporting 47%, 23%, and 39%, respectively. These results highlight the positive impact of supplementation in physiologically compromised individuals and that mixed populations tend to dilute the benefit. An increased risk of falls associated with high baseline levels was also detected, suggesting that this risk should likewise be minimized [14]. This systematic review also highlighted the ongoing controversies surrounding international recommendations for VitD supplementation, beyond its association with falls. One major debate since 2011 concerns the threshold of serum 25(OH)D used to define deficiency and initiate supplementation. The Institute of Medicine (IOM) considers levels below 12 ng/mL (<30 nmol/L) as deficient, whereas other scientific societies recommend treatment at levels below 20 ng/mL (<50 nmol/L) [9,10]. In addition, there have been warnings about significant differences in serum 25(OH)D levels among populations and ethnic groups, which must be considered when establishing supplementation policies. Consequently, there is no consensus on a universal threshold value below which supplementation should be offered. In 2021, the US Preventive Services Task Force concluded that there was insufficient evidence to recommend routine screening for VitD deficiency in asymptomatic adults. The prevalence of marked deficiency (25[OH]D < 12 ng/mL) varies substantially by race and ethnicity, with higher rates among non-Hispanic Asian (8%), non-Hispanic Black (18%), and Hispanic (6%) populations compared with non-Hispanic White populations (2%) [43]. Another controversy concerns the exponential increase in requests for serum 25(OH)D measurements in public health services. This trend reflects social pressure from multiple sectors, which has prompted these services to implement control mechanisms and establish clear criteria regarding the populations in which such testing should be performed [44,45,46].

Concerning the duration of follow-up, long-term studies permit a better assessment of efficacy and safety, although the risk of dropouts and changes in health status may affect results. The meta-analysis demonstrated a non-significant increase in the risk of falls following supplementation >24 months. With respect to shorter studies (≤12 months), there are limitations in sustained changes, although they are useful for vulnerable populations. The meta-analysis showed a non-significant reduction of 4%, although one short study with combined calcium and vitamin D supplementation reported a significant reduction in falls of 27% [25].

### Strengths and Limitations

Among the strengths of this meta-analysis is the fact that only community-dwelling participants were considered, while other meta-analyses included institutionalized individuals with greater vulnerability due to lower exposure to the sun, more comorbidities that may influence the risk of falls, and less mobility, making them more prone to sarcopenia and thus further increasing their risk [39]. Therefore, despite including fewer RCTs than other meta-analyses and reporting a non-significant result, this study presents greater homogeneity in its findings. Similarly, the sub-analysis with 700–1000 UI/day and a non-significant favorable trend contributes added value bearing in mind the scarcity of literature on community-dwelling populations in this dose range.

This meta-analysis has several limitations. First, certain limitations of the individual studies included may have influenced the overall results. Some studies had relatively small sample sizes [15,24,25,27,28,29,30], particularly in subgroup analyses, which reduces the weight of their findings in the pooled estimates. In contrast, three studies contributed substantially to the overall results [26,31,32]: for example, Kärkkäinen et al., showing a non-significant 2% reduction in falls, accounted for 39.9% of the total weight; Waterhouse et al., with a non-significant 2% increase, accounted for 33.9%; and Bischoff-Ferrari et al., with a non-significant 3% increase, contributed 14.6%. These influential studies partly explain the overall trend toward a non-significant reduction in falls.

Other limitations of the included studies relate to the underreporting of falls due to annual or biannual assessments, recall bias, or incomplete self-reporting [15,26]. This may be a potential source of bias; however, it is unlikely to have affected our findings, as no significant effect of VitD supplementation on fall risk was observed. Additionally, Bischoff-Ferrari et al., 2006, and Kärkkäinen et al. combined VitD with calcium, without administering calcium to the control group, limiting the ability to isolate the effect of VitD on falls.

Finally, this review could not stratify results based on baseline VitD insufficiency, which, according to the literature, might confer a greater benefit [1,2,6]. In addition, although 9 of the 10 studies administered cholecalciferol, the most active and efficient form of elevating serum levels of VitD, one used ergocalciferol without categorizing the effect [24]. Its exclusion would have reduced the number of studies, while its inclusion, being methodologically sound, contributed to the value of this meta-analysis focused on a community-dwelling population.

## 5. Conclusions

The results of this meta-analysis indicate that supplementation with VitD is not effective in reducing the risk of falls in community-dwelling adults >65 years. Nor were beneficial effects observed in the subgroup analyses by sex, dosage, duration, treatment frequency, and baseline VitD levels. More RCTs are needed which specifically assess these aspects to improve understanding of the benefits. It may be that multifactorial approaches are required, combining supplementation with other interventions such as strength and balance exercises to achieve a significant reduction in falls.

## Figures and Tables

**Table 1:** Description of the randomized clinical trials included in the meta-analysis.

Author, Year	Country	Follow-Up Period (Months)	n	Age (Years)	Women (%)	Study Site	Outcome Measure	Baseline Levels of VitD (ng/mL ± SD)	Intervention			Control
									Supplementation	Dose (IU)	Frequency	
Bischoff-Ferrari, 2006 [15]	Switzerland	36	445	≥65	55.2	Community-living people	Falls every 6 months	29.8 ± 13.43	Oral VitD3 + calcium citrate (500 mg/day)	700	Daily	Placebo
Prince, 2008 [24]	Australia	12	302	70–90	100.0	Community-living people	Falls every 6 weeks	17.6 ± 5.05	Oral VitD2 + calcium citrate (1000 mg/day)	1.000	Daily	Calcium citrate (1000 mg/day)
Pfeifer, 2009 [25]	Germany/Austria	12	242	≥70	78.9	Community-living people	Falls every 2 months	21.8 ± 7.2	Oral VitD3 + calcium carbonate (1000 mg/day)	800	Daily	Calcium carbonate (1000 mg/day)
Kärkkäinen, 2010 [26]	Finland	12	3139	≥65	100.0	Community-living people	Falls annually	19.86 ± 7.3	Oral VitD3 + calcium carbonate (1000 mg/day)	800	Daily	Placebo/nothing
Glendenning, 2012 [27]	Australia	9	686	≥70	100.0	Community-living people	Falls every 3 months	26.32 ± 9.8	Oral Vit D3	150.000	3 monthly	Placebo
Uusi-Rasi, 2015 [28]	Finland	24	370	70–80	100.0	Homebound people	Falls monthly	26.88 ± 7.15	Oral VitD3	800	Daily	Placebo
Houston, 2015 [29]	USA	5	64	≥65	72.0	Homebound people	Falls monthly	20.9 ± 11.5	Oral VitD3	100.000	Monthly	Placebo
Appel, 2021 [30]	USA	24	647	≥70	43.6	Community-living people	Falls monthly	22.12 ± 5.8	Oral VitD3	1.000	Daily	Vitamin D3 (200 IU/day)
Waterhouse, 2021 [31]	Australia	60	15,416	≥60	45.9	Community-living people	Falls monthly	31 ± 10.08	Oral VitD3	60.000	Monthly	Placebo
Bischoff-Ferrari, 2022 [32]	Switzerland, Germany, Austria, France, Portugal	36	1900	≥70	61.7	Community-living people	Falls every 6 months	22.4	Oral VitD3 and/or omega3 (1000 mg/day)	2.000	Daily	Placebo

SD = Standard Deviation; IU = International Units; D3 = cholecalciferol; D2 = ergocalciferol.

**Table 2:** Summary table of the subgroup analyses presented in Figure 2.

Subgroup Analyses	Number of Studies	Number of Participants	Effect Size (RR/HR/OR)	95% CI	*p*-Value	Heterogeneity I^2^
Figure 2a. Overall	10	23,211	0.99	0.95–1.03	0.16	31.3%
Figure 2b. Women	7	13,509	0.97	0.92–1.02	0.19	31.20%
Figure 2c. Men	3	9702	1.08	0.98–1.2	0.44	0.00%
Figure 2d. ≤1000 IU doses	6	5145	0.96	0.90–1.02	0.14	39.50%
Figure 2e. >1000 IU doses	4	18,066	1.02	0.96–1.09	0.46	0.00%
Figure 2f. Daily doses	7	7045	0.97	0.92–1.03	0.14	37.20%
Figure 2g. Intermittent doses	3	16,166	1.02	0.95–1.09	0.28	22.50%
Figure 2h. Normal baseline VitD levels	5	5287	0.98	0.92–1.04	0.74	0.00%
Figure 2i. ≤12 months	5	4433	0.96	0.90–1.02	0.06	56.20%
Figure 2j. >12 months	5	18,778	1.01	0.96–1.07	0.66	0.00%
Figure 2k. Without calcium supplementation	6	19,083	1.02	0.96–1.08	0.66	0.00%

## Data Availability

The datasets are available in the Supplementary Material and upon reasonable request from the corresponding author.

## Supplementary Materials

The following supporting information can be downloaded at https://www.mdpi.com/article/10.3390/jcm14176117/s1. Table S1. Search methods for identification of studies; Table S2. Determination of falls in the different studies included in the meta-analysis; Table S3. Quality of the meta-analysis articles; Table S4. The reason for exclusion for each study; Table S5. Results on the effectiveness of VitD supplementation for the prevention of falls observed in each study; Table S6. Sensitivity analysis results when removing one study from (a) all studies, (b) women, and (c) men. Leave one out. Figure S1. Graphical representation of the results of falls of the 10 studies included in the meta-analysis. Funnel plot includes the common-effects and random-effects results; Figure S2. Graphical representation of the trim and fill of the 10 studies included in the meta-analysis plus 2 filled studies. Forest plot. Figure S3. Graphical representation of the trim and fill of the 10 studies included in the metanalysis and 2 filled studies. Funnel plot.

## Abbreviations

The following abbreviations are used in this manuscript:

CI	Confidence Interval
HR	Hazard Ratio
OR	Odds Ratio
RCTs	Randomized Clinical Trials
RR	Relative Risk
VitD	Vitamin D
