# Supplementary material for: Efficacy of Vitamin D Supplementation on the Risk of Falls Among Community-Dwelling Older Adults: A Systematic Review and Meta-Analysis
Source: J Clin Med. 2025 Aug 29;14(17):6117. doi: 10.3390/jcm14176117 (PMC12429313; doi:10.3390/jcm14176117)

SUPPLEMENTARY MATERIALS

Table S1. Search methods for identification of studies.

Database	Date	Search strategy	Final results
PUBMED	April 2023	((("Vitamin D"[Mesh] OR "Ergocalciferols"[Mesh] OR "Vitamin D Deficiency"[Mesh] OR "Cholecalciferol"[Mesh] OR (Vitamin D) OR colecalciferol OR ergocalciferols) OR (((("Calcium"[Mesh]) OR "Calcium Metabolism Disorders"[Mesh]) OR "Calcium, Dietary"[Mesh]) OR (Calcium OR (calcium dietary) OR (calcium metabolism disorders) OR (calcium deficiency)))) OR ((((("Food"[Mesh] OR "Diet, Food, and Nutrition"[Mesh] OR "Eating"[Mesh]) OR ("Diet"[Mesh] OR "Diet, Healthy"[Mesh] OR "diet therapy" [Subheading])) OR "Nutritional Status"[Mesh]) OR "Elder Nutritional Physiological Phenomena"[Mesh]) OR ((Diet, food and nutrition OR nutritional status OR elderly nutrition OR diet, healthy OR diet OR staple food OR food)))) AND (("Accidental Falls"[Mesh]) OR ((fall* OR accidental falls OR fall prevention))).	452
	January 2024		11
	May 2024		2
	August 2024		8
		Results	473
EMBASE	April 2023	#1: 'vitamin d'/exp OR 'vitamin d' OR 'ergocalciferol'/exp OR ergocalciferol OR 'vitamin d deficiency'/exp OR 'vitamin d deficiency' OR 'colecalciferol'/exp OR colecalciferol OR 'calcium'/exp OR calcium OR 'electrolyte disturbance'/exp OR 'electrolyte disturbance' OR 'calcium intake'/exp OR 'calcium intake' OR 'calcium deficiency'/exp OR 'calcium deficiency' OR 'food'/exp OR food OR 'diet'/exp OR diet OR 'nutrition'/exp OR nutrition OR 'eating'/exp OR eating OR 'healthy diet'/exp OR 'healthy diet' OR 'diet therapy'/exp OR 'diet therapy' OR 'nutritional status'/exp OR 'nutritional status' OR 'geriatric nutrition'/exp OR 'geriatric nutrition' OR 'staple food'/exp OR 'staple food' AND 'falling'/exp OR falling OR 'fall prevention'/exp OR 'fall prevention' #2 'randomized controlled trial'/de AND [aged]/lim	362
	January 2024		27
	May 2024		10
	August 2024		5
		Results	404
Total Databases			877

Table S2. Determination of falls in the different studies included in the meta-analysis.

Author, year	Supplementation	Follow-up outcome (months)	History of falls	Yes/No Drop improvement
Bischoff-Ferrari, 2006	Vitamin D3 (700 IU/day) + calcium citrate (500mg/day)	Every 6 months	They send a letter after each fall	Yes, 46% in women and 65% in less active women
Prince, 2008	Vitamin D2 (1000 IU/day) + calcium citrate (1000mg/day)	Every 6 weeks	Diary record of possible falls	Yes, patients with and history of falling and vitamin D insufficiency living in sunny climates
Pfeifer, 2009	Vitamin D3 (800 IU/day) + calcium carbonate (1000mg/day)	Every 2 months	Diary	Yes, but was higher with supplementation calcium
Kärkkäinen, 2010	Vitamin D3 (800 IU/day) + calcium carbonate (1000mg/day)	Annual		Yes, only for those who need medical attention
Glendenning, 2012	Vitamin D3 (150.000 IU/3 monthly)	Every 3 months	Diary	No improvement of falls
Uusi-Rasi, 2015	Vitamin D3 (800 IU/daily)	Monthly	Diary	No
Houston, 2015	Vitamin D3 (100000 IU/monthly)	Monthly		Yes, but only in rate of falls in adjusted analyses
Appel, 2021	Vitamin D3 (1000 IU/daily)	Monthly	Diary	No
Waterhouse, 2021	Vitamin D3 (60000 IU(monthly)	Every 3 months	Diary	No, and a possible increased risk in people with BMI
Bischoff-Ferrari, 2022	Vitamin D3 (2000 IU/day) and/or omega 3 (1000 mg/day)	Every 6 months	They send a letter after each fall	Yes, but a modest benefit

Table S3. Quality of the meta-analysis articles.

Author, year	Initial participants	Final participants	Loss to follow-up (%)	Blind	Hidding assignment
**Bischoff-Ferrari, 2006**	445	445	0	Double-blind	Yes
**Prince, 2008**	302	302	0	Double-blind	Yes
**Pfeifer, 2009**	242	242	0	Double-blind	Yes
**Kärkkäinen, 2010**	3432	3139	8.53	None	Yes
**Glendenning, 2012**	686	686	0	Double-blind	Yes
**Uusi Rasi 2015**	409	370	9.53	Double-blind	Yes
**Houston, 2015**	68	64	5.88	Single blind	Yes
**Appel, 2021**	688	647	5.95		Probably yes
**Waterhouse, 2021**	21315	15416	27.67	Double-blind	Yes
**Bischoff-Ferrari, 2022**	2157	1900	11.91	Double-blind	Yes

Table S4. The reason for exclusion for each study

Number	Author/year	Title	Reason for exclusion
1	Porthouse, 2005	Randomized controlled trial of supplementation with calcium and cholecalciferol (vitamin D3) for prevention of fractures in primary care	1
2	Sanders, 2010	Annual high-dose oral vitamin D and falls and fractures in older women: a randomized controlled trial	1
3	Cummings, 2010	A single annual, high dose of oral vitamin D increased falls and fractures in older women: Commentary	1
4	Lips, 2010	Once-weekly dose of 8400 IU vitamin D (3) compared with placebo: effects on neuromuscular function and tolerability in older adults with vitamin D insufficiency	1
5	Geusens, 2010	Effect of high-dose once-yearly bolus of oral vitamin D on falls and fractures in older women	1
6	Pekkarinen, 2013	Hip fracture prevention with a multifactorial educational program in elderly community-dwelling Finnish women	1
7	Patil, 2016	Cost-effectiveness of vitamin D supplementation and exercise in preventing injurious falls among older home-dwelling women: findings from and RCT	1
8	Hung, 2016	High-dose vitamin D supplementation may lead to increased risk of falls	1
9	Hsieh, 2023	Factors associated with falls in older adults: a secondary analysis of a 12-month randomized controlled trial	1
10	Kiehn, 2009	Vitamin D supplement intake in elderly people	2
11	Janssen, 2010	Muscle strength and mobility in vitamin D-insufficient female geriatric patients: a randomized controlled trial on vitamin D and calcium supplementation	2
12	Bolland, 2010	Vitamin D insufficiency and health outcomes over 5 y in older women	2
13	Hansen, 2015	Treatment of vitamin D insufficiency in postmenopausal women: a randomized clinical trial	2
14	Scragg, 2019	Overview of results from the vitamin D assessment (ViDA) study	2
15	Aloia, 2019	Vitamin D and falls in older African American women: the PODA randomized clinical trial	2
16	Dhaliwal, 2018	The relationship of physical performance and osteoporosis prevention with vitamin D in older African Americans (PODA)	3
17	Cai, 2022	The effects of vitamin D supplementation on frailty in older adults at risk for falls	3
18	Feng, 2021	Comprehensive interventions including vitamin D effectively reduce the risk of falls in elderly osteoporotic patients	4
19	Uusi-Rasi, 2019	Serum 25-hydroxyvitamin D levels and incident falls in older women	5
20	Wanigatunga, 2021	The effects of vitamin D supplementation on types of falls	5
21	Pfeifer, 2006	The role of vitamin D in the treatment of osteoporosis in the elderly	6
22	Schoënmarkers, 2013	Vitamin D supplementation in older adults (VDOP): study protocol for randomized intervention with monthly oral dosing with 24,000 IU or 48,000 IU of vitamin D3	6
23	López-Torres, 2014	Effect of calcium and vitamin D in the reduction of falls in the elderly: A randomized trial versus placebos	6
24	Yousefian, 2015	Falls increased on recommended doses of vitamin D in elderly women	6
25	Rubinova, 2017	Vitamin D and falls in older African american women-the PODA trial	6
26	Smith, 2017	Medium doses of daily vitamin D decrease falls and higher doses or daily vitamin D3 increase falls: A randomized clinical trial	6
27	Aspray, 2019	Randomized controlled trial of vitamin D supplementation in older people to optimize bone health	6
28	Ozsoy-Unubol, 2021	The effect of vitamin D and exercise on balance and fall risk in postmenopausal women: a randomized controlled study	6
29	Saito, 2021	Effect of eldecalcitol on muscle function and fall prevention in Japanese postmenopausal women: a randomized controlled trial	6
30	Guralnik, 2022	Effects of vitamin D on physical function: results from the STURDY trial	6
31	Schrack, 2023	The association of vitamin D supplementation and serum vitamin D levels with physical activity in older adults: results from a randomized trial	6
32	Bischoff-Ferrari, 2021	DO-HEALTH: Vitamin D3-Omega3-Home exercise- healthy aging and longevity trial - design of a multinational clinical trial on healthy aging among European seniors	6
33	Bischoff-Ferrari, 2008	Additive benefit of higher testosterone levels and vitamin D plus calcium supplementation in regard to fall risk reduction among older men and women	7
34	Leboff, 2020	Vitamin D and Omega-3 Trial (VITAL): Effects of vitamin D supplements on risk of falls in the US populations	7

Reason for exclusion: 1 = wrong setting 2 = wrong patient population 3= wrong intervention 4= wrong dosage 5= subanalyses of the initial sample 6= non statistical data 7= non stratified data

Table S5. Results on the effectiveness of VitD supplementation for the prevention of falls observed in each study.

Author,year		OR (IC INF-IC SUP)	OR	IC inf	IC sup	beta	BETA inf	BETA sup	EE inf	EE sup
**Bischoff-Ferrari, 2006**	Men	0.93 (0.50-1.72)	0.9	0.5	1.72	-0.0725707	-0.6931472	0.54232429	0.3166207	0.3137219
**Bischoff-Ferrari, 2006**	Women	0.54 (0.30-0.97)	0.5	0.3	0.97	-0.6161861	-1.2039728	-0.0304592	0.2998912	0.2988403
**Bischoff-Ferrari, 2006**	All	0.77 (0.51-1.15)	0.8	0.51	1.15	-0.2613648	-0.6733446	0.13976194	0.2101938	0.2046565
**Prince, 2008**	Women	0.61 (0.37-0.99)	0.6	0.37	0.99	-0.4942963	-0.9942523	-0.0100503	0.2550796	0.2470643
**Pfeifer, 2009**	All	0.73 (0.54-0.96)	0.7	0.54	0.96	-0.3147107	-0.6161861	-0.040822	0.153814	0.1397392
**Kärkkäinen, 2010**	Women	0.98 (0.92 - 1.05)	1	0.92	1.05	-0.0202027	-0.0833816	0.04879016	0.0322341	0.0352004
**Glendenning, 2012**	Women	1.06 (0.75 - 1.49)	1.1	0.75	1.49	0.05826891	-0.2876821	0.39877612	0.1765056	0.1737282
**Uusi Rasi, 2015**	Women	1.08 (0.77 - 1.52)	1.1	0.77	1.52	0.07696104	-0.2613648	0.41871033	0.1726152	0.1743619
**Houston, 2015**	All	0.48 (0.19-1.19)	0.5	0.19	1.19	-0.7339692	-1.6607312	0.17395331	0.4728378	0.4632258
**Appel, 2021**	All	0.94 (0.76 - 1.15)	0.9	0.76	1.15	-0.0618754	-0.2744368	0.13976194	0.1084497	0.1028762
**Waterhouse, 2021**	Men	1.05 (0.94-1.18)	1.1	0.94	1.18	0.04879016	-0.0618754	0.16551444	0.056462	0.0595532
**Waterhouse, 2021**	Women	1.00 (0.89-1.13)	1	0.89	1.13	0	-0.1165338	0.12221763	0.059456	0.0623559
**Waterhouse, 2021**	All	1.02 (0.95 - 1.10)	1	0.95	1.1	0.01980263	-0.0512933	0.09531018	0.0362734	0.0385243
**Bischoff-Ferrari, 2022**	Men	1.20 (1.00-1.45)	1.2	1	1.45	0.18232156	0	0.37156356	0.0930212	0.096552
**Bischoff-Ferrari, 2022**	Women	0.92 (0.81-1.04)	0.9	0.81	1.04	-0.0833816	-0.210721	0.03922071	0.0649691	0.0625522
**Bischoff-Ferrari, 2022**	All	1.03 (0.92-1.14)	1	0.92	1.14	0.0295588	-0.0833816	0.13102826	0.0576227	0.0517701

Table S6. Sensitivity analysis results when removing one study from (a) all studies, (b) women and (c) men. Leave one out.

Author, year	beta.fixed	se.fixed	pval.fixed	beta.random	se.random	pval.random
**Bischoff-Ferrari, 2006**	-0.009232709	0.02225627	0.6782617	-0.009245653	0.02227531	0.6780960
**Prince, 2008**	-0.008370917	0.02221616	0.7063266	-0.008375657	0.02222403	0.7062673
**Pfeifer, 2009**	-0.005639556	0.02236449	0.8009130	-0.005642751	0.02237278	0.8008749
**Kärkkäinen,2010**	-0.006718245	0.02856958	0.8140894	-0.033056610	0.04173335	0.4283076
**Glendenning, 2012**	-0.013141529	0.02230740	0.5557869	-0.013177766	0.02234308	0.5553303
**Uusi Rasi, 2015**	-0.013493311	0.02231356	0.5453694	-0.013496897	0.02231700	0.5453245
**Houston, 2015**	-0.010445033	0.02215659	0.6373417	-0.010445506	0.02215719	0.6373356
**Appel, 2021**	-0.009838647	0.02261222	0.6634876	-0.009879611	0.02266712	0.6629409
**Waterhouse, 2021**	-0.028472115	0.02722700	0.2956853	-0.028473489	0.02722843	0.2956874
**Bischoff-Ferrari, 2022**	-0.019192192	0.02394415	0.4228191	-0.019202619	0.02395248	0.4227285

(a)

Author, year	beta.fixed	se.fixed	pval.fixed	beta.random	se.random	pval.random
Bischoff-Ferrari, 2006	-0.02811222	0.02678852	0.2939880	-0.02811233	0.02678878	0.2939909
Prince, 2008	-0.02765681	0.02682963	0.3026201	-0.02765831	0.02683362	0.3026657
Kärkkäinen, 2010	-0.05047320	0.04122982	0.2208799	-0.05047334	0.04123003	0.2208810
Glendenning, 2012	-0.03487326	0.02699080	0.1963423	-0.03490822	0.02703003	0.1965440
Uusi Rasi, 2015	-0.03540598	0.02700172	0.1897735	-0.03541388	0.02701023	0.1898144
Waterhouse, 2021	-0.04021004	0.02955979	0.1737365	-0.04025163	0.02959987	0.1738747
Bischoff-Ferrari, 2022	-0.02258175	0.02926140	0.4402776	-0.02259880	0.02931774	0.4408116

(b)

Author, year	beta.fixed	se.fixed	pval.fixed	beta.random	se.random	pval.random
Bischoff-Ferrari, 2006	0.08508202	0.05102712	0.09543721	0.09332463	0.06339699	0.14100274
Waterhouse, 2021	0.16016798	0.09275474	0.08420533	0.16016798	0.09275474	0.08420533
Bischoff-Ferrari, 2022	0.04381512	0.05895330	0.45735003	0.04381512	0.05895330	0.45735003

(c)

Figure S1. Graphical representation of the results of falls of the 10 studies included in the meta-analysis. Funnel plot includes the common-effects and random-effects results.

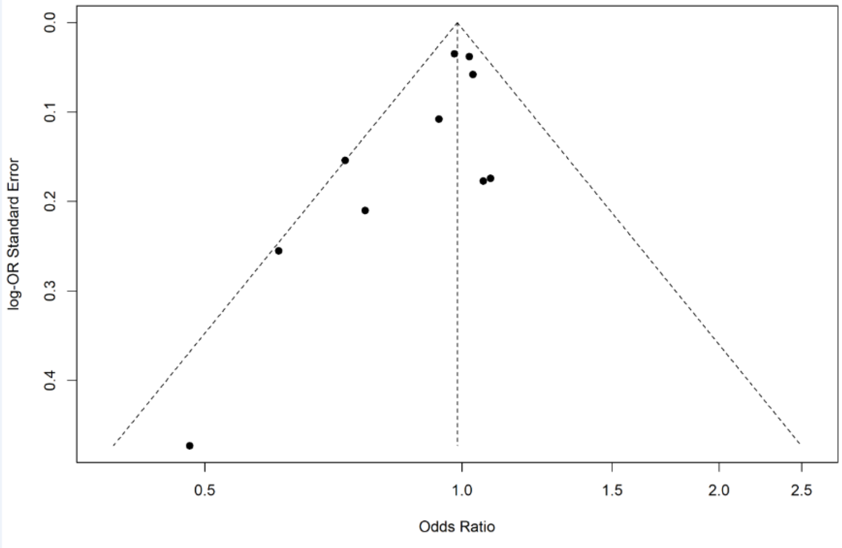

Figure S2. Graphical representation of the trim and fill of the 10 studies included in the meta-analysis plus 2 filled studies. Forest plot.

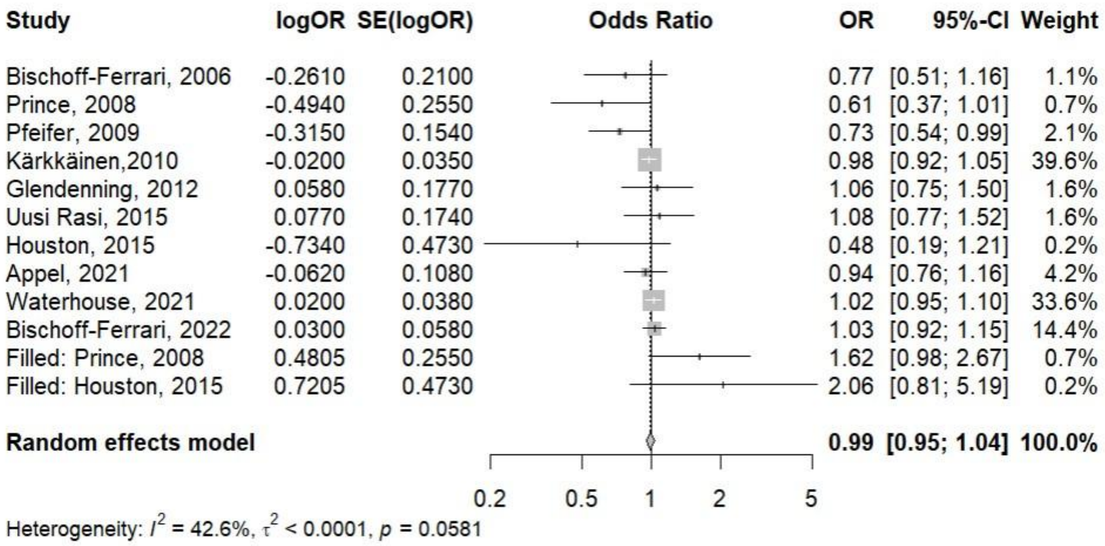

Figure S3. Graphical representation of the trim-and-fill of the 10 studies included in the meta-analysis and 2 filled studies. Funnel plot.

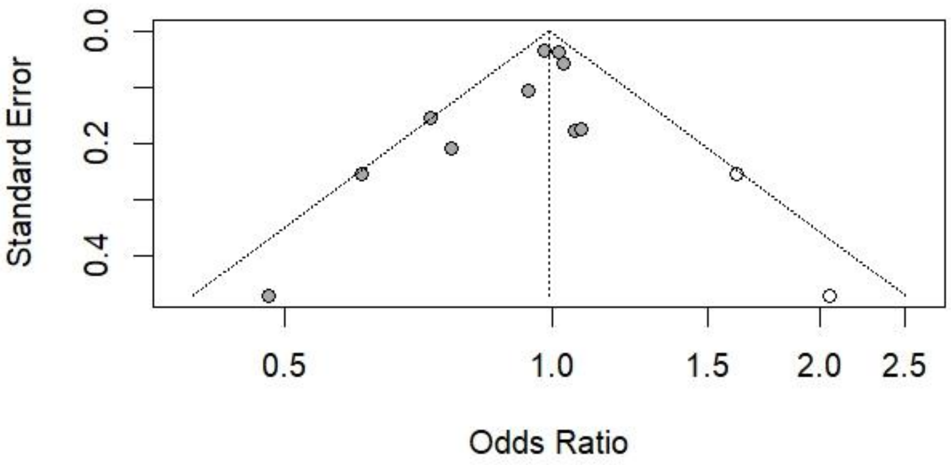

Supplement: Supplementary file 1 [file jcm-14-06117-s001.zip › jcm-3767799-supplementary.pdf]
